# Motorized Power Spiral Enteroscopy (MSE): Is Routine Bougienage of the Upper Esophageal Sphincter (UES) Necessary?

**DOI:** 10.7759/cureus.52342

**Published:** 2024-01-15

**Authors:** Alok Kumar Singh, Siddharth Srivastava, Ujjwal Sonika, Sanjeev Sachdeva, Payila Aneesh, Ajay Kumar, Barjesh C Sharma, Ashok Dalal

**Affiliations:** 1 Gastroenterology and Hepatology, G.B. Pant Hospital, New Delhi, IND

**Keywords:** bougie dilatation, enteroscope, bougienage, upper esophageal sphincter, motorized power spiral enteroscopy

## Abstract

Introduction

Wire-guided bougienage of the upper esophageal sphincter (UES) was performed routinely before per-oral motorized power spiral enteroscopy (MSE). In the present study, we aimed to answer the clinical question of whether routine bougienage of UES is required.

Methods

This was a retrospective study that included 20 patients who underwent antegrade spiral enteroscopy for various indications. The feasibility and safety of anterograde MSE without prior bougie dilatation of the upper esophageal sphincter were assessed. The technical success rate (TSR), diagnostic yield, and adverse events (AEs) were also assessed.

Results

In 16 out of the 20 patients, a spiral enteroscope was taken directly across UES into the esophagus without a prior bougie dilatation. The spiral enteroscope could not be negotiated across UES only in one patient, and bougie dilatation was done. The technical success rate was 100%. The diagnostic yield was 80%. Four patients reported AEs.

Conclusions

MSE had a good technical success rate and diagnostic yield. Routine dilatation of the UES before the procedure may be unnecessary.

## Introduction

It has never been easy to evaluate the small bowel endoscopically. For small-bowel examination, single-balloon enteroscopy (SBE) and double-balloon enteroscopy (DBE) systems are suitable choices [1,2]. The spiral enteroscopy was introduced in 2008. It was necessary to manually rotate a spiral overtube to pleat the small bowel over the enteroscope. [3]. The most recent technology that operates on the same principles as spiral enteroscopy is the novel motorized power spiral enteroscopy (MSE) [4]. In a recent study, MSE performed better than SBE. It allowed a deeper examination of the small intestine. The duration of the procedure was also shorter [5]. MSE also had a comparatively shorter learning curve [6]. In theory, by accelerating the process, facilitating faster insertion, and simplifying the technique, MSE addressed the drawbacks faced by endoscopists in evaluating small bowel [7]. Although the technique has been withdrawn from the market due to safety concerns, it may come with modifications in the near future.

Before each procedure, bougie dilatation of the upper esophageal sphincter (UES) using a Savary bougie of 18-20 mm size was carried out [8-10]. There are no studies supporting the need for dilating UES routinely before each MSE procedure. In the present study, we aimed to find out whether routine bougienage of UES is required before peroral MSE.

## Materials and methods

A retrospective analysis of patients undergoing MSE procedures at the Department of Gastroenterology from January 2022 to May 2023 was conducted for this study at the G.B. Pant Institute of Postgraduate Medical Education and Research (GIPMER), New Delhi, India. Retrospective data collection was done from clinical records and proformas, which were filled during each MSE procedure. This was a retrospective data analysis of all the patients who underwent MSE at our centre. It was planned to recruit more patients but MSE was withdrawn from the market. Hence data of all the patients who underwent MSE were analyzed. Written and informed consent was obtained from each patient before the procedure. As it was a retrospective data analysis, institutional ethical clearance was waived. The data was calculated in numbers and percentages.

Inclusion criteria included age greater than 18 years of either gender and patients with suspected small bowel pathology based on clinical presentation, imaging, or capsule endoscopy.

Exclusion criteria included contraindications for endoscopy such as poor general condition of the patient, respiratory distress, patients in shock or altered sensorium, inability to provide written and informed consent, patients with esophageal or gastric varices, coagulopathy, pregnancy, prior abdominal surgery, and inability to tolerate sedation.

There has already been a description of the MSE procedure [4]. Olympus Medical Systems Corporation (Tokyo, Japan) produced the tool, which was a motorized spiral enteroscope. Every procedure was done in the left lateral position under general anesthesia and with nasotracheal intubation. Each patient had an upper gastrointestinal endoscopy to rule out the stricture or web of the esophagus. Initially, a few patients underwent bougie dilation of the esophagus up to 20 mm to make the transit of spiral tubes across the UES easier. In subsequent procedures, no prior bougienage was attempted; instead, we directly went ahead with a spiral enteroscope.

The primary objective of the study was to assess the feasibility of antegrade MSE without prior UES dilatation. Secondary objectives were technical success rate (TSR), diagnostic yield, and adverse events (AEs). When the enteroscope successfully passes through the ligament of Treitz, it is referred to as technical success. The percentage of procedures that produce positive results that might be used to explain the clinical symptoms is known as the diagnostic yield.

There were two types of adverse events (AEs): minor and major. Minor adverse effects included a sore throat, mild retrosternal pain or discomfort, nausea, vomiting, or discomfort in the abdomen that goes away in less than 48 hours, and slight mucosal oozing from a superficial mucosal rupture. Significant bleeding, perforation, pancreatitis, aspiration pneumonitis, or an extended hospital stay due to a procedure-related complication were among the major adverse events.

## Results

Our study included 20 patients who underwent antegrade spiral enteroscopy for various indications. Eight out of twenty patients were male. The mean (±Standard Deviation) age of the study population was 39 (±15) years. The most common indications for the procedure were obscure gastrointestinal bleeding and partial small bowel obstruction. Two patients had jejunal growth, and one had chronic diarrhea with anemia (Table 1).

**Table 1:** Baseline characteristics of the study population N=sample size, %=percentage

Patient characteristics	No. of patients (%) (N=20)
Sex	
Male	8 (40%)
Female	12 (60%)
Indications of procedure	
Obscure gastrointestinal bleed	9 (45%)
Partial small bowel obstruction	8 (40%)
Jejunal mass	2 (10%)
Chronic diarrhoea	1 (5%)
Bougie dilatation	
Yes	4 (20%)
No	16 (80%)

In the first four patients out of 20, UES dilatation was done before attempting MSE. In the remaining 16 patients, it was attempted to negotiate the spiral enteroscope directly across the UES into the esophagus without a prior bougie dilatation. In only one patient out of 16, the spiral enteroscope could not be negotiated across UES. Bougie dilatation of UES was done, following which the spiral enteroscope could be negotiated across UES. In all the remaining 15 patients, a spiral enteroscope could be easily negotiated across the UES.

100% of patients achieved the technical success rate. The overall diagnostic yield was 80%. Four patients reported adverse events. All of them had minor AEs. Two patients had abdominal discomfort lasting less than 48 hours, and two patients had sore throats lasting less than 48 hours (Table 2).

**Table 2:** Secondary objectives N = sample size, % = percentage

Objectives	No. of patients (%) (N=20)
Technical success rate	20 (100%)
Diagnostic yield	16 (80%)
Adverse events	4 (20%)

Different diagnoses can be appreciated in Table 3.

**Table 3:** Different diagnoses made by motorized power spiral enteroscopy n = No. of patients with a diagnosis, % = percentage

Diagnoses	No. of patients (%) (n=16)
Stricture (with or without ulcers)	5 (31.25%)
Ulcers	4 (25%)
Erythema and erosions	4 (25%)
Polyp	1 (6.25%)
Mass	1 (6.25)
Vascular lesion (angioectasia)	1 (6.25%)

## Discussion

Motorized spiral enteroscopy was a very useful technique for the evaluation of small-bowel pathology. It had a shorter learning curve and was gradually gaining popularity, but was sadly withdrawn from the market due to some complications.

There is no evidence supporting bougie dilatation of UES in every case of antegrade MSE. The decision regarding this was usually left to the endoscopist's discretion. Our study included 20 patients who underwent an antegrade spiral for various indications. Serial bougie dilatation of the esophagus was done up to 20 mm in the first four patients. In subsequent procedures, no prior bougie dilatation was attempted; instead, we directly went across UES with a spiral enteroscope. In 16 out of 20 patients, a spiral enteroscope could be taken across UES into the oesophagus without a prior bougie dilatation. In only one patient, a spiral enteroscope could not be negotiated across UES, and bougie dilatation was required. In all remaining patients, a spiral enteroscope could be easily negotiated across the UES. Thus, the present study shows that dilatation of UES could be avoided in a majority of patients, which would cut short the procedure time and avoid unnecessary dilatation-related complications.

In our study, technical success was achieved in 100% of patients. In another study, TSR was 95.55% with the antegrade route [9]. The technical success rate was 92.85% in a retrospective analysis employing MSE for 61 small bowel disease patients [8]. TSR was 96% for anterograde enteroscopy, according to a recent meta-analysis [11]. The overall diagnostic yield was 80% in our study. The diagnostic yield was 85.18% in a recent study [9]. In the other two studies, the diagnostic yield was 70% and 74.2%, respectively [8,12]. A recent meta-analysis suggested a cumulative diagnostic success rate of 78% (735/959), which was similar to our study [11]. A Chinese meta-analysis revealed an overall diagnostic yield for DBE of 68.1% [13]. Another study found that the pooled diagnostic yields for DBE and SBE were 64.4% and 53.9%, respectively [14]. Thus, spiral enteroscopy scored both over SBE and DBE. Four (20%) patients reported adverse events in our study. All had minor AEs. A study from India reported AEs in 24.5% of patients. All patients had minor adverse events [8]. In a prospective observational study, severe AEs occurred in 2.3% of patients [15]. In a recent meta-analysis, AEs were described in 17% of cases (151/959). Most AEs were minor (16%) [11].

Our study had a few limitations. The sample size was small. It was a retrospective study with no comparative arm. It was a single-center study. A prospective study with large sample size and a comparative arm would have been better to prove with statistical significance that one strategy (non-bougienage of UES) was better than the other (bougienage). Also, we did not assess anthropometric parameters, patients built, or endoscopic level of training.

## Conclusions

MSE had a good technical success rate and diagnostic yield. Antegrade MSE without routine UES dilatation is feasible and safe. This could avoid unnecessary dilatation-related complications and also reduce the procedure time.
